# Sustainable Synthesis of Biomass-Based Carbon Quantum Dots for Selective Fluorescent Recognition of Cr^3+^ and In Vitro Antioxidant Applications

**DOI:** 10.3390/molecules31101585

**Published:** 2026-05-09

**Authors:** Yu Zhang, Yinying Zhang, Min Liu, Lifen Meng

**Affiliations:** 1 School of Chemical Engineering, Guizhou University of Engineering Science, Bijie 551700, China; 2 Guizhou Key Laboratory for Germplasm Innovation and Resource-Efficient Utilization of Dao-di Herbs, Bijie Institute of Traditional Chinese Medicine, Bijie 551700, China

**Keywords:** biomass–carbon quantum dots, fluorescence sensing, Cr^3+^ detection, environmental water analysis, antioxidant activity

## Abstract

The development of cost-effective, eco-friendly, sensitive, and efficient analytical platforms for the monitoring of metal ions holds profound practical value. In this work, *edible fungus* carbon quantum dots (*Ef-CQDs*) are synthesized via a facile hydrothermal route using *edible fungus* as a green carbon precursor, and a novel fluorescence sensing strategy is established for the rapid and selective detection of Cr^3+^ in environmental water matrices. Systematic optical investigations revealed that the as-prepared *Ef-CQDs* displayed outstanding selectivity toward Cr^3+^ over other coexisting metal ions. Meanwhile, the *Ef-CQDs* exhibited considerable scavenging activity toward hydroxyl radicals and DPPH radicals, endowing them with favorable antioxidant performance. When applied for Cr^3+^ determination in real environmental water samples, the proposed *Ef-CQDs* achieved satisfactory spiked recoveries ranging from 95.2% to 100.6%. This study provided a promising and sustainable approach for the green, rapid, and reliable monitoring of Cr^3+^ in complex aqueous environments.

## 1. Introduction

In recent years, with the rapid development of industrialization and urbanization, the problem of residual heavy metal ions (Ba^2+^, Pb^2+^, Fe^3+^, Cu^2+^, etc.) in water bodies, soils, and food environments is becoming increasingly serious [1,2]. Their toxicity and excessive accumulation pose a serious threat to the ecological environment and human health. For example, Ba^2+^ can cause acute poisoning, causing irritation to the human gastrointestinal tract, nausea, vomiting, abdominal pain, and diarrhea, as well as affecting soil microbial activity, inhibiting plant growth and development, leading to adverse conditions such as slow growth and even death of plant leaves [3]. Other metal ions also cause varying degrees and directions of damage to the human body and environment [4]. The discharge of metal-ion-containing wastewater in industry and agriculture has long been a serious environmental problem, and even trace amounts of metal ions can cause significant harm to human health [5,6,7,8]. Therefore, it is important to develop an efficient, sensitive, simple, and low-cost method for detecting metal ions.

Carbon quantum dots (CQDs) are a new type of fluorescent carbon nanomaterial that has emerged in recent years [9]. Due to their low production cost, small size, good biocompatibility, strong hydrophilicity, rich surface functional groups, and strong and stable photoluminescence, CQDs are widely used in the field of sensing [10,11]. Therefore, owing to their unique luminescent properties, CQDs have been expanded for applications in biology and medicine. Typical CQDs are zero-dimensional carbon-dominated nanomaterials composed of sp^2^/sp^3^ carbon skeletons and abundant functional groups and polymer chains, with sizes generally less than 10 nm [12,13]. The abundant surface functional groups/polymer chains, such as carboxyl, hydroxyl, and amine groups, endow CQDs with excellent water solubility, enabling them to easily composite with other materials without phase separation [14,15]. In addition, the diverse functional groups make CQDs readily amenable to modification with various organic or polymer molecules, making them an ideal choice for a variety of fluorescent sensors [16,17,18].

There are various methods for preparing CQDs, including chemical synthesis, the template method, and the hydrothermal method [19,20]. Among them, the hydrothermal method has become an important part of preparing carbon dot solutions due to its simple operation, environmental friendliness, and ease of regulation. This study used foods as a carbon source to prepare CQDs through the hydrothermal method, which can not only achieve effective utilization of resources but also endow CQDs with unique fluorescence properties [21,22]. Research has shown that CQDs can achieve highly sensitive detection of heavy metal ions through fluorescence quenching or enhancement effects. Compared to organic dyes containing cadmium or lead and certain traditional quantum dots, these CQDs not only possess excellent optical properties but also have advantages such as lower toxicity, ease of surface modification, cost-effectiveness, and excellent biocompatibility [23,24,25].

Although traditional methods for detecting metal ions in the environment, such as high-performance liquid chromatography, the fluorescence method, atomic absorption spectroscopy, etc., although have high sensitivity, the pre-treatment is complex, the cycle is long, and the operation is cumbersome [26,27,28]. Moreover, the current instrument prices are high, the initial purchase costs are high, and the mid-term operation requires professional personnel for maintenance [29]. The high cost of consumables such as chromatography columns and flow meters greatly limits their application prospects [30]. The preparation of CQDs based on natural ingredients not only conforms to the concept of green chemistry but also reduces preparation costs, providing a benign and sustainable solution for environmental testing.

This study used CQDs prepared from *edible fungus* (*Ef-CQDs*) ingredients to detect metal ions, which showed good recovery and stability in actual environmental water samples and soil samples. It provided a green and economical new method for rapid and efficient detection of environmental pollutants, and has the potential to be widely applied in the field of environmental monitoring.

## 2. Results and Discussion

### 2.1. Characterization of Ef-CQDs

**Figure 1:**
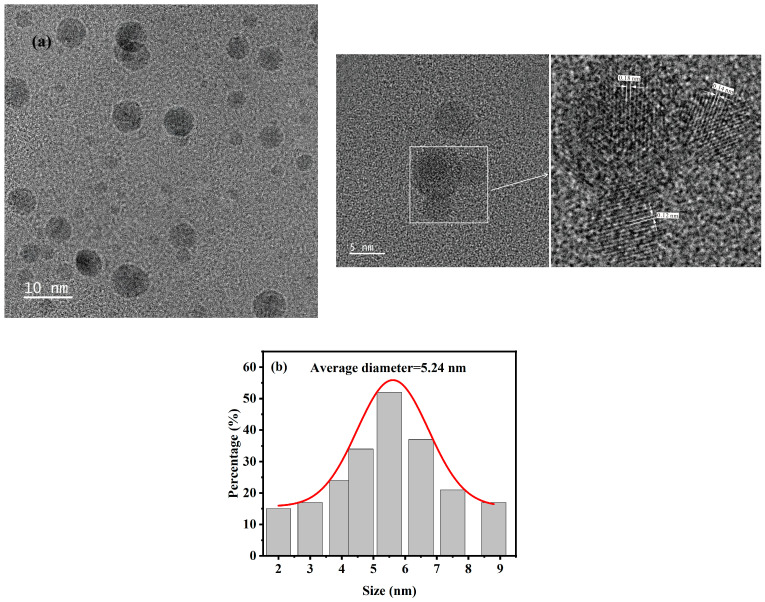
(**a**) TEM of *Ef-CQDs* (the image on the right: a high-resolution transmission electron microscopy image), (**b**) Particle size distribution of *Ef-CQDs*.

Characterization and analysis of the morphological characteristics of *Ef-CQDs* were performed using TEM. As shown in Figure 1, *Ef-CQDs* exhibit uniformly distributed spherical nanostructures with a relatively uniform particle size distribution. The illustration shows a high-resolution transmission electron microscopy (HRTEM) image, from which it can be observed that the lattice spacing of *Ef-CQDs* measured was 0.18 nm, corresponding to the 100 interface of graphene [31]. According to the analysis in Figure 1b, the particle size distribution range of synthesized *Ef-CQDs* was between 2 and 8.8 nm, and the calculated average particle size was 5.24 nm.

**Figure 2:**
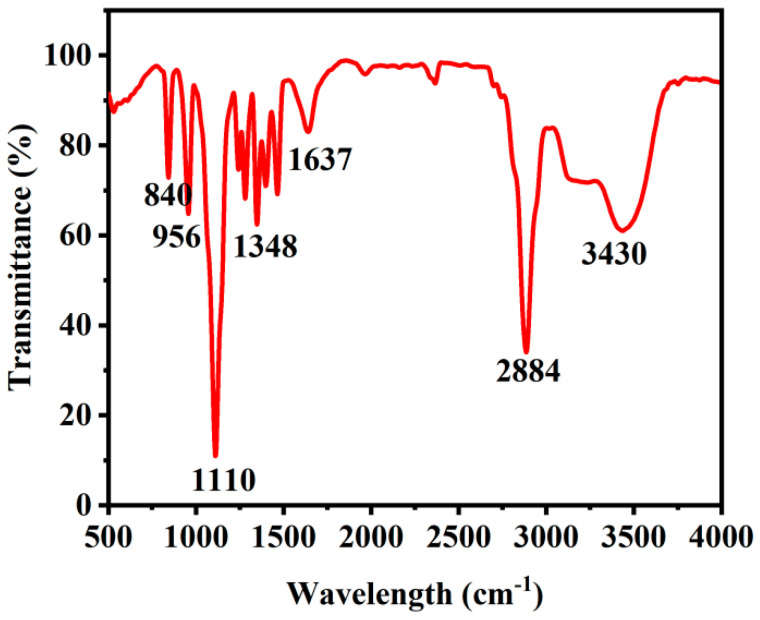
FTIR spectra of the *Ef-CQDs*.

The surface functional groups of the *Ef-CQDs* were characterized using Fourier Transform Infrared Spectroscopy (FTIR) (Figure 2). The spectral results show that the characteristic absorption peak at 840 cm^−1^ typically refers to the out of plane bending vibration of =C-H on triple or tetra substituted double bonds; at 956 cm^−1^ is the characteristic absorption peak of trans disubstituted alkenes; the characteristic absorption peak at 1110 cm^−1^ could be attributed to the bending vibration mode of C-N and C-O bonds; the absorption peak at 1348 cm^−1^ corresponds to the C-H bending [32]; the characteristic peak at 1637 cm^−1^ is the stretching vibration of C=O bonds; and the absorption peak at 2884 cm^−1^ originates from the stretching vibration of C-H bonds [32]. The characteristic peak at 3430 cm^−1^ was attributed to the stretching vibration of N-H bonds [33].

**Figure 3:**
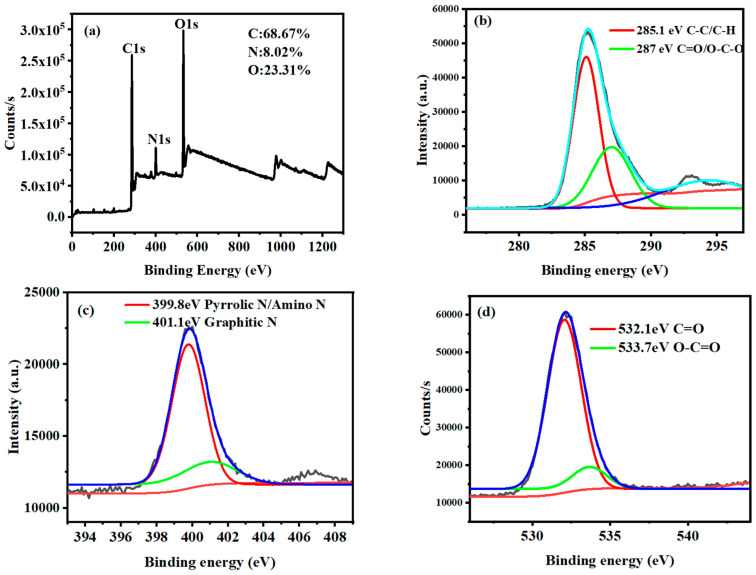
XPS full spectrum analysis of *Ef-CQDs* (**a**), high-resolution XPS spectra of C1s (**b**), N1s (**c**), and O1s (**d**).

The elemental composition and valence states of the *Ef-CQDs* were characterized using X-ray photoelectron spectroscopy (XPS, Figure 3). The *Ef-CQDs* contained elements such as O, N, and C, which were consistent with the elemental types of the reaction precursors. As shown in Figure 3a, the XPS full spectrum of *Ef-CQDs* shows characteristic peaks of C1s, N1s, and O1s at binding energies of approximately 285 eV, 400 eV, and 532 eV, respectively. The proportions of each element were C: 68.67%, N: 8.02%, and O: 23.31%, which confirmed that *Ef-CQDs* were N-doped carbon-based nanomaterials.

Peak fitting was performed on the high-resolution spectrum of C1s (Figure 3b), and two characteristic peaks were observed. The peak at 285.1 eV corresponded to the C-C/C-C-H bond, which was the sp^2^/s^3^ carbon skeleton of CQDs. The peak at 287 eV corresponded to the C=O/O-C-O bond, indicating the presence of O-containing functional groups such as carbonyl and ether bonds on the material surface, which provided a structural basis for its hydrophilicity and biocompatibility.

The N1s high-resolution spectrum (Figure 3c) was fitted with two peaks, with the peak at 399.8 eV belonging to pyrrole type N/amino N, mainly in N-doped form, and the peak at 401.1 eV belonging to graphite type N, which accounted for a relatively low proportion overall. This doping structure could effectively regulate the electronic structure and optical properties of CQDs, which was the key to their excellent fluorescence performance.

The O1s high-resolution spectrum (Figure 3d) was fitted with two peaks, with the peak at 532.1 eV corresponding to the C=O bond and the peak at 533.7 eV corresponding to the O-C=O bond. These O-containing functional groups were shown to endow materials with good hydrophilicity and reactivity, serving as active sites for fluorescent probes or biomaterials.

Therefore, the XPS characterization results indicated that *Ef-CQDs* are N-doped carbon-based nanomaterials, and their surfaces were rich in O-containing functional groups such as carbonyl and carboxyl groups. Their elemental composition and chemical bonding environment provided structural basis for subsequent optical performance and application research.

**Figure 4:**
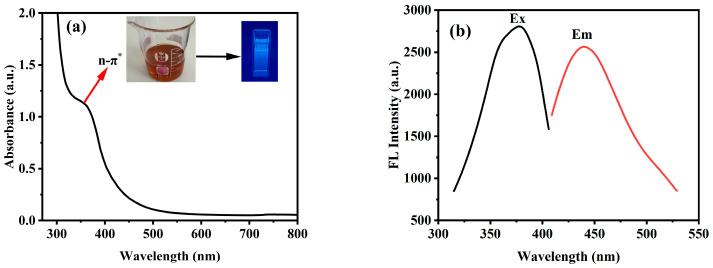
(**a**) UV-visible spectra and (**b**) fluorescence emission and excitation spectra of *Ef-CQDs*.

In Figure 4a of the UV-visible absorption spectrum of *Ef-CQDs*, the black curve shows a clear absorption shoulder peak at 300–400 nm, corresponding to the n → π* transition, which was the electronic transition of oxygen-containing/nitrogen-containing functional groups. In Figure 4b of the fluorescence spectrum, the black curve represented the excitation spectrum with an optimal excitation wavelength of 377 nm, and the red curve represented the emission spectrum with an optimal emission wavelength of approximately 440 nm, exhibiting typical blue fluorescence emission with excitation wavelength-dependent characteristics, which was closely related to the surface defect state structure of CQDs. According to Section 3.3, the QY of the *Ef-CQDs* was determined and calculated by the instrument to be 21.68%.

### 2.2. Fluorescence Performance Stability

**Figure 5:**
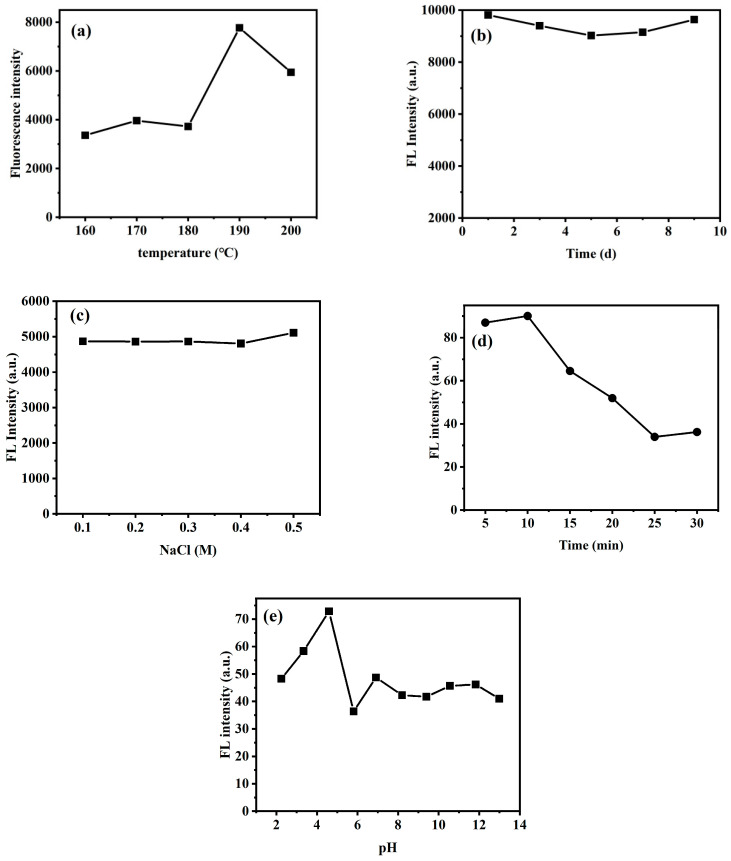
The influence of preparation temperature (**a**), storage time (**b**), and ionic strength (**c**) on the fluorescence intensity of *Ef-CQDs*; the influence of reaction time (**d**) and pH (**e**) on fluorescence quenching efficiency (F/F_0_).

Results of the optimal preparation temperature for *Ef-CQDs* are shown in Figure 5a. In the temperature range of 160–180 °C, the fluorescence intensity of *Ef-CQDs* is at a low level and fluctuates gently, indicating that the carbon skeleton and surface fluorescence emission center have not yet fully formed within this temperature range. When the temperature rises to 190 °C, the fluorescence intensity sharply increases and reaches its peak, indicating that 190 °C is the optimal reaction temperature for this system. At this time, the surface defect states and conjugated structures of CQDs are most complete, and the fluorescence performance is optimal. Further increasing the temperature to 200 °C resulted in a decrease in fluorescence intensity, which may be due to excessive carbonization of the carbon skeleton or decomposition of surface functional groups caused by high temperature, thereby damaging the fluorescence emission center.

At the optimal preparation temperature of 190 °C, the fluorescence intensity changes of *Ef-CQDs* within 0–10 days were investigated to evaluate their storage stability. The results are shown in Figure 5b. Overall, the fluorescence intensity remained at a high level within 10 days without significant attenuation. This result indicates that *Ef-CQDs* have excellent long-term storage stability and can meet the needs of batch preparation, long-term storage, and actual sample testing.

In order to understand the interference of common ions on the fluorescence performance of *Ef-CQDs* in complex environments, the effects of different concentrations of NaCl addition on the fluorescence intensity of *Ef-CQDs* was studied, and the results are shown in Figure 5c. When the amount of NaCl added increased from 0.1 to 0.5 M, there was a slight decrease in fluorescence intensity. This result indicates that the presence of NaCl has a weak effect on the fluorescence performance of *Ef-CQDs*, and the material can still maintain excellent fluorescence stability in high-salt environments with good resistance to ion interference, providing a basis for its detection of Cr^3+^ in complex environments such as actual water samples. 

At the optimal preparation temperature of 190 °C, the effect of reaction time on the fluorescence performance of *Ef-CQDs* for detecting Cr^3+^ was further investigated, as shown in Figure 5d. Within 0–5 min of reaction, the fluorescence intensity rapidly increases and reaches its peak, indicating a rapid interaction between the two, reflecting the rapid response characteristics of the probe. As the reaction time prolongs (5–25 min), the fluorescence intensity slowly decreases, which may be due to subsequent structural changes or fluorescence quenching processes in the bound complex. The fluorescence intensity tends to stabilize within 25–30 min, and the system enters a dynamic equilibrium state. Therefore, 5 min is suitable for rapid qualitative detection, 25 min is suitable for quantitative analysis, and the corresponding reaction time can be selected according to experimental needs in the future.

The effect of different pH values on the fluorescence performance of *Ef-CQDs* for detecting Cr^3+^ was investigated, as shown in Figure 5e. The results showed that the fluorescence intensity first increased with the increase of pH and reached its peak at pH 5, indicating that the interaction between *Ef-CQDs* and Cr^3+^ was the strongest and the fluorescence response was the most sensitive under this condition. At pH 6, the fluorescence intensity significantly decreased, possibly due to an increase in pH that altered the form of Cr^3+^ or the surface charge of *Ef-CQDs*, weakening the interaction between the two. At pH 7–14, the fluorescence intensity remains stable at a low level, indicating that *Ef-CQDs* have a weak response to Cr^3+^ in neutral to alkaline environments. Therefore, pH 5 is the optimal pH for detecting Cr^3+^.

### 2.3. Selective and Anti-Interference Properties of Ef-CQDs

**Figure 6:**
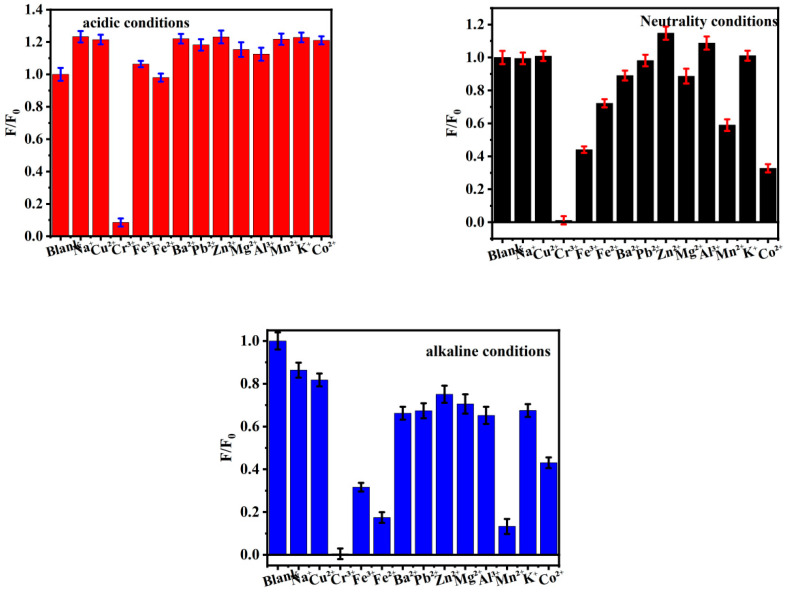
The influence of different metal ions on the fluorescence performance of *Ef-CQDs* under acidic, neutral, and alkaline conditions.

In order to evaluate the practical application potential of *Ef-CQDs* as fluorescent probes, the fluorescence quenching efficiency (F/F_0_) of *Ef-CQDs* to various common metal ions (Na^+^, K^+^, Cr^3+^, Fe^3+^, Cu^2+^, etc.) under neutral, acidic, and alkaline conditions were investigated. The results are shown in Figure 6. As shown in the figures, only Cr^3+^ significantly quenched the fluorescence of *Ef-CQDs*, indicating that the *Ef-CQDs* had a strong specific recognition ability for Cr^3+^. In contrast, other heavy metal ions such as Na^+^, K^+^, Mg^2+^, Ca^2+^, Pb^2+^, Zn^2+^, etc., have minimal effects on the fluorescence intensity of *Ef-CQDs*. So, *Ef-CQDs* exhibit excellent selective detection performance for Cr^3+^ in complex environment systems and have good anti-interference ability.

### 2.4. Sensitivity of Ef-CQD Detection for Cr^3+^

**Figure 7:**
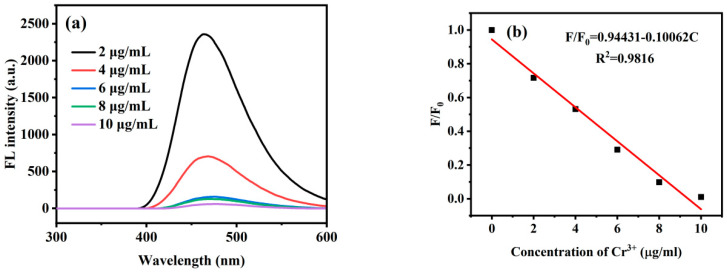
(**a**) The fluorescence spectra of *Ef-CQDs* at different Cr^3+^ concentrations, (**b**) The linear correlation between Cr^3+^ concentration and F_0_/F in the range of 0–10 μg/mL.

Sensitivity was an important factor in measuring the detection performance of prepared *Ef-CQDs*. In order to further investigate the sensitivity of *Ef-CQDs* to Cr^3+^ detection, the fluorescence spectra of *Ef-CQDs* in the presence of different concentrations of Cr^3+^ were statistically analyzed, as shown in Figure 7a. Figure 7b shows the relationship curve between F/F_0_ and Cr^3+^ concentration in the range of 0–10 μg/mL, where F represented the fluorescence intensity with the addition of Cr^3+^ and F_0_ represented the fluorescence intensity of the blank control group. This figure illustrates the relationship between Cr^3+^ concentration and fluorescence quenching of *Ef-CQDs*.

As shown in Figure 7a, with the increase of Cr^3+^ concentration in the solution (2–10 μg/mL), there was no significant change in the emission peak position of *Ef-CQD* solution at 440 nm, but the fluorescence intensity gradually decreased with the increase in Cr^3+^ concentration, indicating that *Ef-CQDs* were sensitive to changes in Cr^3+^ concentration. This suggested that *Ef-CQDs* could be used as a fluorescence sensor for detecting Cr^3+^ ion concentration. Figure 7b shows that there was a good linear relationship between Cr^3+^ concentration and fluorescence effect when the Cr^3+^ concentration was between 0 and 10 μg/mL; F/F_0_ = 0.94431 − 0.10062C, R^2^ = 0.9816. Based on the standard calculation method [34], the limit of detection (LOD) was determined to be 29.8 ng/mL, indicating the high sensitivity of the proposed sensing platform. In summary, the *Ef-CQDs* had high sensitivity and selectivity for sensing Cr^3+^ and excellent performance as fluorescent probes for Cr^3+^.

### 2.5. Detection Mechanism

**Figure 8:**
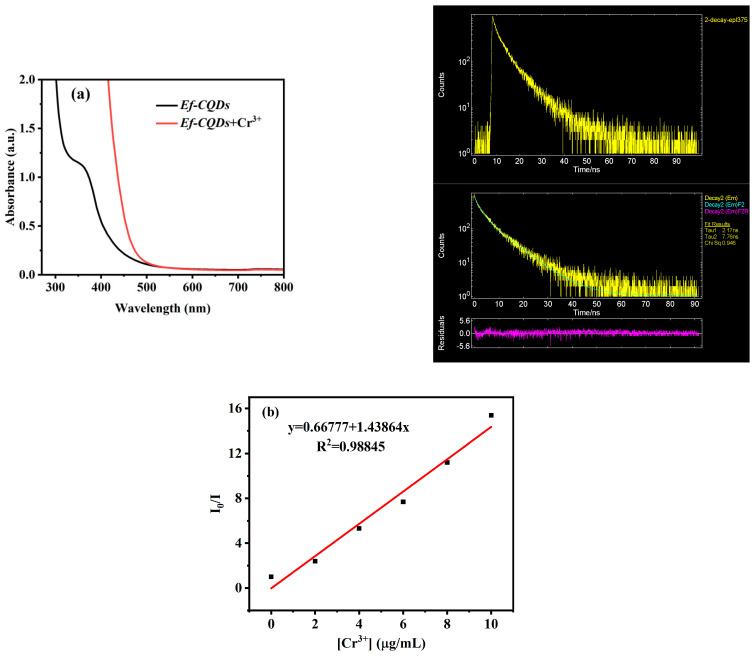
(**a**) UV-visible absorption spectra of Cr^3+^, *Ef-CQDs* before and after adding Cr^3+^; the fluorescence lifetime of *Ef-CQDs* before and after adding Cr^3+^, (**b**) Stern–Volmer plot of fluorescence quenching of *Ef-CQD* solution system by Cr^3+^.

To further investigate the interaction mechanism between *Ef-CQDs* and Cr^3+^, the UV-vis spectra before and after adding Cr^3+^ to *Ef-CQD* solution were examined, as shown in Figure 8a. The addition of Cr^3+^ significantly changed the UV-vis absorption of *Ef-CQDs*, with the absorption peak at 350 nm. However, after adding Cr^3+^, this absorption peak disappeared and no new absorption peak appeared, indicating that some chemical reactions occurred between *Ef-CQDs* and Cr^3+^, resulting in the formation of compounds without absorption characteristics. Therefore, it was speculated that the quenching mechanism was static quenching [35,36].

On the other hand, static quenching was caused by the combination of fluorescent groups and quenchers to generate non-fluorescent substances, and the presence of quenchers did not change the fluorescence lifetime of fluorescent groups. Dynamic quenching was caused by the collision between fluorescent groups and quenchers, resulting in a decrease in fluorescence intensity; the presence of quenchers could shorten the fluorescence lifetime [37,38]. To further explore its quenching mechanism, the fluorescence lifetime of *Ef-CQDs* before and after the addition of Cr^3+^ was measured as shown in Figure 8b. The results showed that there was no significant change in the average lifetime of *Ef-CQDs* before and after the addition of Cr^3+^, which were 7.76 ns and 7.91 ns, respectively. The above tests all indicate that the fluorescence quenching of *Ef-CQDs* by Cr^3+^ was static quenching.

In order to further explore the quenching mechanism, the Stern–Volmer equation (1) was used for analysis [39]:

*I*_0_/*I* = 1 + *K_sv_* [Cr^3+^] (1)

where *I*_0_ and *I* are the fluorescence intensities of *Ef-CQDs* in the absence and presence of Cr^3+^, [Cr^3+^] is the concentration of Cr^3+^, and *K_sv_* is the Stern–Volmer quenching constant. A well-fitted linear Stern–Volmer plot was observed within the detection range (0–10 μg/mL, Figure 8b). Generally, dynamic quenching originates from excited-state collision, while static quenching is derived from the formation of non-fluorescent ground-state complexes. Combined with the irreversible binding interaction between *Ef-CQDs* and Cr^3+^, the fluorescence quenching in this system is mainly controlled by the static quenching process.

### 2.6. Actual Sample Testing Results

In order to evaluate the detection performance of the *Ef-CQDs* in complex water matrices, spiked recovery experiments were conducted on tap water, river water, and domestic sewage. The tap water samples were collected from the laboratory of Guizhou University of Engineering Science, the river water samples were collected from the Liucang river in Bijie city, and the domestic sewage samples were collected from the residential area of Guizhou University of Engineering Science. The experimental results showed that the spiked recovery rates of the *Ef-CQDs* in different water samples were 95.2–100.6%, and the relative standard deviations (RSDs) were between 2.5 and 4.8% (Table 1). These results indicated that the *Ef-CQDs* had good detection stability and reliability in complex aqueous matrices and could effectively recognize Cr^3+^ under controlled conditions. Although the background concentration of Cr^3+^ in water samples was low, making it difficult to directly detect its natural content, spiked recovery experiments had validated the applicability of the *Ef-CQDs* in different types of water samples, indicating its potential application value in water quality analysis and trace detection.

### 2.7. Analysis of Two Free Radical Scavenging Activities of Ef-CQDs

**Figure 9:**
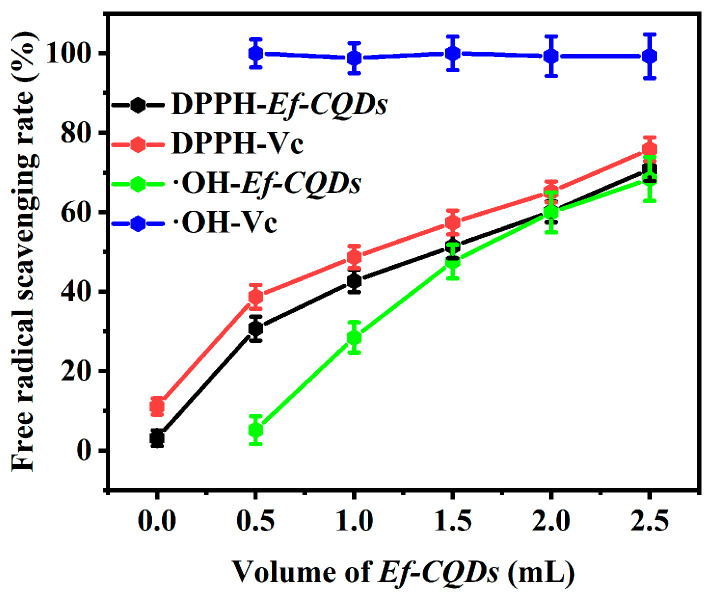
DPPH and **·**OH radical scavenging activities of *Ef-CQDs*.

In order to investigate the antioxidant properties of *Ef-CQDs*, Vc was used as a positive control to determine the scavenging rate of different volumes of *Ef-CQDs* on hydroxyl radicals (**·**OH). The results are shown in Figure 9. The positive control Vc showed a stable clearance rate of 98–100% within the range of 0.5–2.5 mL, demonstrating efficient and stable free radical scavenging ability, verifying the reliability of the experimental system. *Ef-CQDs* exhibited a significant concentration-dependent scavenging effect. As the volume of *Ef-CQDs* increased from 0.5 mL to 2.5 mL, the **·**OH scavenging rate of *Ef-CQDs* gradually increased from about 5–65%, demonstrating a typical concentration-dependent effect. Although the clearance rate of *Ef-CQDs* was lower than that of the positive control Vc, their enhanced clearance activity with increasing dosage also indicated that *Ef-CQDs* had good antioxidant potential.

Figure 9 shows the DPPH radical scavenging activity of *Ef-CQDs* against Vc. As the volume of *Ef-CQDs* increased, their clearance rates showed an upward trend, exhibiting a clear dose-dependent characteristic. At all volumes, the clearance activity of Vc was higher than that of *Ef-CQDs*. When the volume was 2.5 mL, the clearance rates of Vc and *Ef-CQDs* reached 75.8% and 70.84%, respectively, and their clearance activities were similar. This also indicated that *Ef-CQDs* had good DPPH radical scavenging potential.

The scavenging trends indicate *Ef-CQDs* possess a higher affinity for **·**OH than DPPH radicals, arising jointly from their surface functional groups and intrinsic electronic structure. XPS-verified abundant C=O, O-C=O, and -OH groups endow *Ef-CQDs* with good aqueous compatibility and sufficient active sites, favoring interfacial contact and electron/hydrogen donation toward water-soluble **·**OH. In contrast, the lipophilic, sterically hindered DPPH exhibits weaker accessibility to surface sites and lower reaction affinity. Meanwhile, the conjugated carbon framework of *Ef-CQDs* provides a delocalized electronic system, facilitating electron transfer and stabilizing unpaired radicals. Therefore, surface oxygenated groups regulate radical accessibility and reaction compatibility, while the electronic structure dominates electron transfer behavior, collectively determining the selective scavenging trend of *Ef-CQDs*.

The divergent scavenging behaviors toward DPPH and **·**OH further underline the unique antioxidant profile of *Ef-CQDs*. Unlike vitamin C, which depends solely on a single hydrogen-donating pathway, *Ef-CQDs* exhibit prominent radical selectivity and multi-pathway scavenging capacity. Rich surface oxygenic functional groups and a delocalized conjugated structure enable *Ef-CQDs* to operate through synergistic hydrogen atom transfer and single electron transfer routes [40]. They present higher affinity toward hydroxyl radicals while retaining effective activity against DPPH.

This dual-mechanism and selective antioxidant feature distinguishes *Ef-CQDs* from conventional small-molecule antioxidants, confers wider environmental adaptability, and greatly reinforces the scientific novelty and application value of this work [34,41,42].

## 3. Experimental Section

### 3.1. Instruments and Reagents

A UV-visible spectrophotometer (Metash, Shanghai Metash Instruments Co., Ltd., UV-5200PC, Shanghai, China), Fluorescence spectrophotometer (Metash, F97PRO, Shanghai Metash Instruments Co., Ltd., Shanghai, China), Fourier transform infrared spectrometer (FTIR, WQF-530A, Beijing Beifen Ruili Analytical Instruments, Beijing, China), transmission electron microscope (TEM, JEOL JEM-F200, Tokyo, Japan), X-ray photoelectron spectrometer (XPS, Thermo Scientific K-Alpha, Waltham, MA, USA), and X-ray diffractometer (XRD, HAOYUAN DX-2700BH, Shanghai, China) were used in this experiment.

*Edible fungus* came from Heilongjiang Weiduobao Food Co., Ltd., Mudanjiang, China, and was purchased from the supermarket next to the school. Ethylenediamine, chromium oxide (Cr^3+^), iron trioxide (Fe^3+)^, ferrous chloride (Fe^2+^), cobalt acetate (Co^2+^), barium nitrate (Ba^2+^), copper sulfate (Cu^2+^), manganese acetate (Mn^2+^), sodium nitrate (Na^+^), lead acetate (Pb^2+^), potassium nitrate (K^+^), aluminum sulfate (Al^3+^), anhydrous magnesium sulfate (Mg^2+^), zinc sulfate heptahydrate (Zn^2+^), diluted hydrochloric acid, sodium hydroxide, sodium chloride, ferrous sulfate, salicylic acid, hydrogen peroxide, DPPH (2-2 biphenyl-1-picrylhydrazino 9), and vitamin C were all from Shanghai Aladdin Reagent Co., Ltd, Shanghai, China.

### 3.2. Preparation of Ef-CQDs

Place the dried *edible fungus* purchased from the supermarket in the sun until it can be crushed by hand, then use a grinder to grind it into a fine powder for later use. Dissolve 0.75 g of *edible fungus* powder in 75 mL of distilled water, add 1.7 mL of ethylenediamine solution, sonicate for 30 min, then place the solution in a PTFE high-pressure reactor and heat it in a 190 °C oven for 10 h. After the reaction is complete, let the solution cool to room temperature. Filter the reaction solution, and filter the filtrate through a 0.45 μm microporous filter head to remove large particles from the reaction solution. Finally, label the solution and store it in the refrigerator for future experimental use.

This study designed a dual-factor gradient experimental scheme. Set five gradients of 6 h, 7 h, 8 h, 9 h, and 10 h in preparation time, and select five gradients of preparation temperature: 160 °C, 170 °C, 180 °C, 190 °C, and 200 °C. By setting different preparation times and temperature gradients, carbon stock solutions under different conditions can be obtained through firing. Further determine the optimal preparation conditions through fluorescence measurement.

### 3.3. Calculation of Quantum Yield

Use quinine sulfate as the standard (0.1 M H_2_SO_4_ as the solvent, QY = 0.54 at 360 nm). The corresponding QY formula of *Ef-CQDs* is shown in Equation (2) [43],

(2) $$Q_{CQDs}=Q_{R}\times \frac{I_{CQDs}}{I_{R}}\times \frac{A_{R}}{A_{CQDs}}\times \frac{\eta_{CQDs}^{2}}{\eta_{R}^{2}}$$

where *Q* is the fluorescence quantum yield, *I* is the integrated area of emission intensity in the fluorescence spectrum, and *A* is the absorbance intensity, where *η* is the refractive index of the sample. The subscript “*R*” represents the reference (quinine sulfate). In order to minimize the reabsorption effect, the absorbance intensity of all tested samples must be kept below 0.1.

### 3.4. Detection of Metal Ions by Ef-CQDs

Mix the prepared *Ef-CQDs* (190 °C, 10 h) with metal ion solutions (Cr^3+^, Fe^3+^, Fe^2+^, Co^2+^, Ba^2+^, Cu^2+^, Mn^2+^, Na^+^, Pb^2+^, K^+^, Al^3+^, Mg^2+^, Zn^2+^) under different pH conditions (pH 2–13) and measure their fluorescence intensity after a period of reaction. Compare the fluorescence intensity of the system after adding metal ions with the blank control group without metal ions and select the pH with the most significant change in fluorescence intensity: take two 5 mL centrifuge tubes and accurately transfer 40 μL of *Ef-CQDs* into the first centrifuge tube using a pipette. Add distilled water to 5 mL, mix thoroughly, and let it stand for 30 min for reaction. Then measure its fluorescence intensity (F_0_, Ex = 377 nm, Em = 440 nm). In the second centrifuge tube, use a pipette to transfer 40 μL of *Ef-CQDs*, then add 1 mL of 1 mg/mL metal ion solution (Cr^3+^, Fe^3+^, Fe^2+^, Co^2+^, Ba^2+^, Cu^2+^, Mn^2+^, Na^+^, Pb^2+^, K^+^, Al^3+^, Mg^2+^, Zn^2+^) and add 1 mL of buffer solution with different pH values. Dilute to 5mL with distilled water, shake thoroughly, and react for 30 min. Measure the fluorescence intensity of the system (Ex = 377 nm, Em = 440 nm).

### 3.5. In Vitro Activity Test

#### 3.5.1. Hydroxyl Radical Scavenging Experiment

Prepare the dried *Ef-CQDs* into 30 mg/mL liquid for later use. Take 0, 0.5, 1, 1.5, 2, and 2.5 mL of the above liquids and place them in 10 mL centrifuge tubes. Add 2 mL of ferrous sulfate solution, 2 mL of salicylic acid solution, and 2 mL of hydrogen peroxide solution to each tube and dilute to the mark. Heat the water bath for 30 min and measure the absorbance A_s_ at 510 nm. Take 2 mL of ferrous sulfate solution, 2 mL of salicylic acid solution, and 2 mL of hydrogen peroxide solution and dilute to the mark. Heat the water bath for 30 min and measure the absorbance A_b_ at 510 nm. Take 0, 0.5, 1, 1.5, 2, and 2.5 mL of *Ef-CQDs* and place them in 10 mL centrifuge tubes, add sulfuric acid to each tube, and measure the absorbance A_s_ at 510 nm. Dilute 2 mL of ferrous solution and 2 mL of salicylic acid solution to the mark, heat the water bath for 30 min, and measure the absorbance A_0_ at 510 nm. Take the same concentration of 30 mg/mL vitamin C solution of the same volume as the diluted solution of *Ef-CQDs* as a control and calculate the scavenging rate. The scavenging rate was calculated using Equation (3):

(3) $$\mathrm{Hydroxyl}\;\mathrm{free}\;\mathrm{radical}\;\mathrm{scavenging}\;\mathrm{rate}\;(\%)\;=\;\frac{1-(A_{s}-A_{0})}{A_{b}}\times 100\%$$

#### 3.5.2. DPPH Radical Scavenging Experiment

Prepare the dried *Ef-CQDs* into 30 mg/mL liquid for later use and mix the above diluents of 0, 0.5, 1, 1.5, 2, and 2.5 mL with 2 mL DPPH solution. Measure the absorbance A_s_ after reacting in the dark for 30 min. Mix 2 mL of ethanol with 2 mL DPPH and measure the absorbance A_b_ after reacting in the dark for 30 min. Take the *Ef-CQDs* diluents of 0, 0.5, 1, 1.5, 2, and 2.5 mL with 2 mL of ethanol as reference samples and measure the absorbance A_0_ at 517 nm. Take 30 mg/mL vitamin C solution of the same volume as the diluted solution of *Ef-CQDs* as a control and calculate the scavenging rate. The scavenging rate was calculated using Equation (4):

(4) $$\mathrm{DPPH}\;\mathrm{free}\;\mathrm{radical}\;\mathrm{scavenging}\;\mathrm{rate}\;(\%)\;=\;\frac{1-(A_{s}-A_{0})}{A_{b}}\times 100\%$$

### 3.6. Analysis of Cr^3+^ Ions in Actual Water Samples

This experiment collected tap water, river water, and domestic sewage to verify the detection effect of *Ef-CQDs* in real water samples. The feasibility and practicality of the fluorescent probe were verified by adding different concentrations of Cr^3+^ ions and calculating the ion recovery rate. The collected river water and lake water samples are filtered through a 0.22 μm filter membrane to remove impurities and insoluble particles.

## 4. Conclusions

This study used *edible fungus* as a natural carbon source and employed a one-step hydrothermal method to green prepare low-cost, water-soluble fluorescent *Ef-CQDs*. TEM, FTIR, XPS, UV-vis, and fluorescence spectroscopy characterization confirmed that the *Ef-CQDs* were spherical nanoparticles with uniform particle size, were rich in oxygen and nitrogen functional groups on the surface, and had typical optical characteristics and blue fluorescence properties. The *Ef-CQDs* responded quickly to Cr^3+^, with outstanding anti-interference ability and recognition selectivity. At the same time, *Ef-CQDs* exhibited good scavenging activity against hydroxyl radicals and DPPH radicals and had potential antioxidant application value. In summary, the *Ef-CQDs* were readily available, environmentally friendly, and had excellent fluorescence and stability performance, which could provide reliable technical reference for heavy metal monitoring in environmental water samples and potential antioxidant application value.

## Figures and Tables

**Table 1:** Detection results of Cr^3+^ in different real water samples (n = 3).

Samples	Spiked (μg/mL)	Detected (μg/mL)	Recovery (%)	RSD (%)
Tap water	0	-	-	-
	5	5.02	100.4	4.1
	10	9.88	98.8	3.2
River water	0	-	-	-
	5	5.03	100.6	4.8
	10	10.02	100.2	2.8
Domestic sewage	0	-	-	-
	5	4.76	95.2	2.7
	10	10.01	100.1	2.5

## Data Availability

No data was used for the research described in the article.

## Abbreviations

CQDs	Carbon quantum dots
Ef-CQDs	Edible fungus carbon quantum dots
HRTEM	High-resolution transmission electron microscopy
FTIR	Fourier Transform Infrared Spectroscopy
XPS	X-ray photoelectron spectroscopy
LOD	Limit of detection
RSDs	Relative standard deviations
TEM	Transmission electron microscope
XRD	X-ray diffractometer
QY	Quantum Yield
